# Extrapulmonary Tuberculosis: A Case of Breast Tuberculosis and Literature Review

**DOI:** 10.7759/cureus.117626

**Published:** 2026-10-08

**Authors:** Ghita Baani, Salma Abrar, Hanane Ikrou, Halloumi Oussama

**Affiliations:** 1 Pulmonology Department, Mohamed VI University Hospital, Laboratoire de Recherche et d’Innovation en Sciences de Santé (LARISS), Faculty of Medicine and Pharmacy of Agadir, Ibn Zohr University, Agadir, MAR; 2 Respiratory Medicine, University Hospital Mohammed VI of Agadir, Agadir, MAR

**Keywords:** breast abscess, breast cancer, breast tuberculosis, case report, extrapulmonary tuberculosis, tuberculosis

## Abstract

Breast tuberculosis is a rare form of extrapulmonary tuberculosis that can closely mimic other breast diseases, particularly pyogenic abscesses and breast cancer, making its diagnosis challenging. We report the case of a 31-year-old woman who presented with a breast swelling initially managed as a pyogenic abscess. Despite antibiotic therapy with amoxicillin/clavulanic acid, no clinical improvement was observed. Given the persistent breast lesion, further investigation was performed, including a percutaneous breast biopsy. Histopathological examination revealed epithelioid and giant cell granulomas with caseous necrosis, findings strongly suggestive of breast tuberculosis. The patient was subsequently managed with antituberculous therapy, with favorable clinical evolution.

This case highlights the diagnostic challenges associated with breast tuberculosis, particularly its clinical and radiological similarity to breast cancer and bacterial breast abscesses. A high index of suspicion is essential, especially in endemic settings or in patients with breast lesions that do not respond to conventional antibiotic treatment. Histopathological examination remains crucial for establishing the diagnosis and avoiding inappropriate or potentially mutilating surgical treatment.

## Introduction

Tuberculosis is an infectious disease caused by *Mycobacterium tuberculosis*, with a predominant pulmonary tropism. Breast tuberculosis represents an unusual extrapulmonary localization and remains a rare condition, even in tuberculosis-endemic countries [1,2]. Although its incidence has decreased in Western countries, breast tuberculosis remains a diagnostic challenge because its clinical and imaging features are nonspecific and may mimic breast cancer [1,3]. The diagnosis relies primarily on microbiological and histopathological findings, including the identification of *M. tuberculosis* and the presence of characteristic epithelioid and giant cell granulomas with caseous necrosis [1,2]. Antituberculous therapy is the cornerstone of treatment, generally consisting of a six-month regimen, with surgical intervention reserved for selected cases such as persistent abscesses or inadequate response to medical treatment [1,2].

We report the case of a 31-year-old woman with no history of pulmonary tuberculosis who presented with a progressively enlarging breast mass over two months, associated with constitutional symptoms and ipsilateral axillary lymphadenopathy. The initial clinical presentation raised concern for breast malignancy, while ultrasonography subsequently suggested a breast abscess. The lesion failed to respond to conventional antibiotic therapy, prompting percutaneous biopsy, which revealed histopathological findings strongly suggestive of breast tuberculosis. The case illustrates the diagnostic challenge of distinguishing breast tuberculosis from other breast conditions, particularly breast cancer and pyogenic abscesses.

## Case presentation

A 31-year-old woman, never previously treated for tuberculosis, with no recent history of tuberculosis exposure and no significant past medical history, presented with a right breast mass that had been gradually increasing in volume for two months, accompanied by feverish sensations, night sweats, and deterioration in her general condition, characterized by profound asthenia and a 4-kg weight loss over two months. Clinical examination revealed a periareolar mass (Figure 1), predominantly occupying the upper outer quadrant of the right breast. The lesion was inflammatory, hard in consistency, fixed to the deeper planes, and associated with nipple retraction and ipsilateral axillary lymphadenopathy. The left breast and the remainder of the somatic examination were unremarkable.

**Figure 1:**
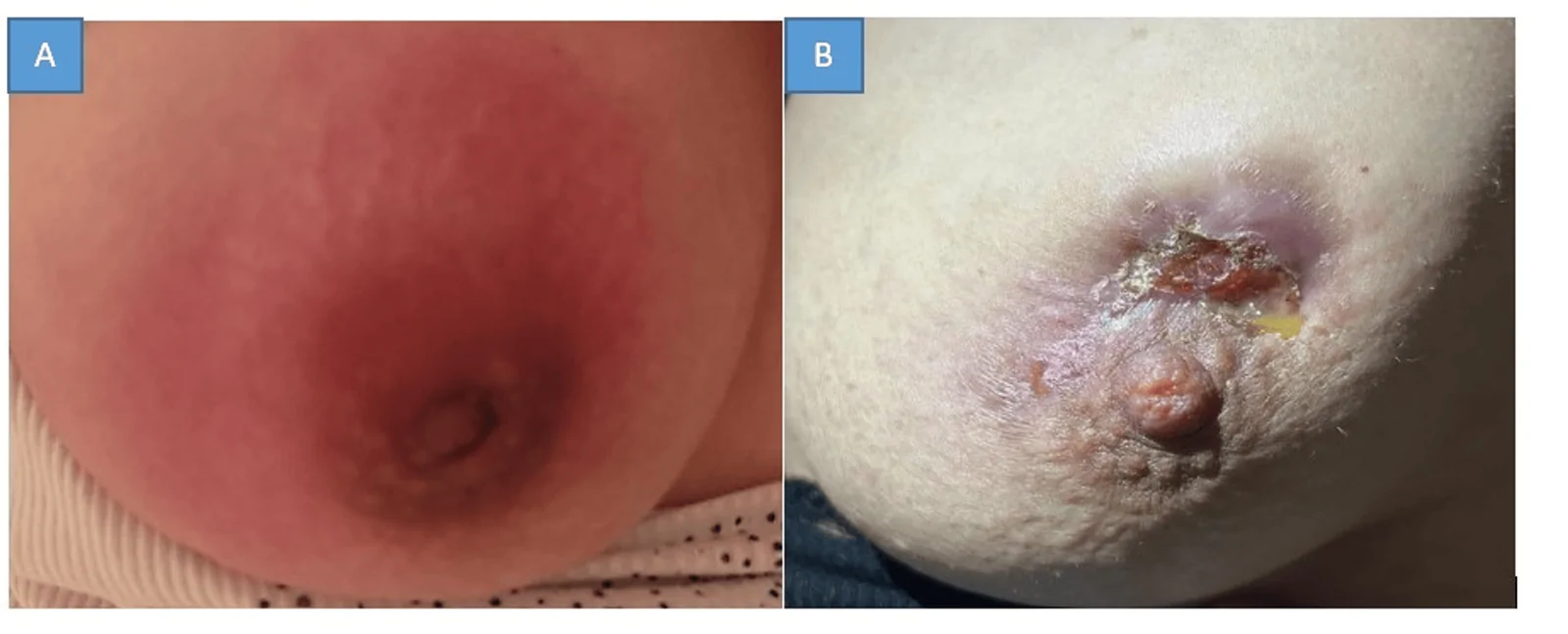
Clinical presentation of breast tuberculosis Clinical evolution of the right breast, from the initial presentation with inflammatory changes and swelling (A) to two months after initiation of anti-tuberculosis treatment and percutaneous drainage (B).

Given the progressive nature and clinical appearance of the breast mass, together with the associated constitutional symptoms and axillary lymphadenopathy, breast malignancy was initially considered and warranted further evaluation.

Mammary ultrasonography was subsequently performed and showed a heterogeneous, hypoechoic retroareolar lesion, poorly vascularized on Doppler, with its long axis parallel to the skin and associated with regular thickening of the overlying skin, suggesting a breast abscess (Figure 2).

**Figure 2:**
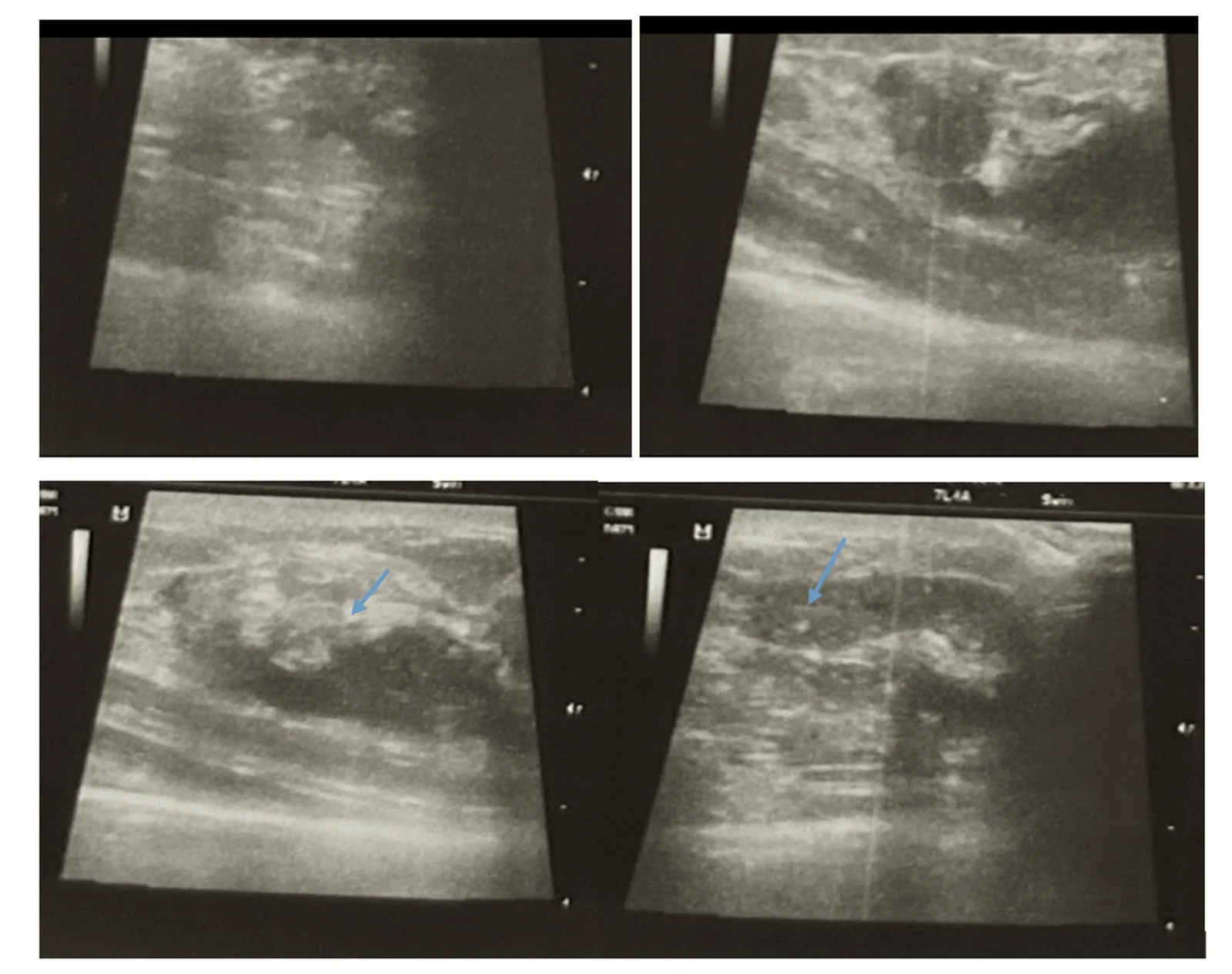
Mammary ultrasonography Breast ultrasonography showed a heterogeneous, hypoechoic retroareolar lesion, poorly vascularized on Doppler, with a long axis parallel to the skin, associated with regular thickening of the overlying skin, suggesting a breast abscess.

Although the imaging findings favored an abscess, they were not specific enough to exclude other underlying conditions, including breast tuberculosis or malignancy.

Biological tests showed an inflammatory syndrome, with a C-reactive protein level of 32 mg/L (normal <5 mg/L), a white blood cell count of 12,790/mm³ (normal 4,000-10,000/mm³), with a predominance of neutrophils, and a lymphocyte count of 2,176/mm³.

Given the clinical, imaging, and biological findings, a pyogenic breast abscess was initially considered the most likely diagnosis. Probabilistic antibiotic treatment with amoxicillin/clavulanic acid at 3 g/day was administered orally for 10 days, without significant clinical improvement.

A percutaneous biopsy with drainage was subsequently performed. Pathological examination revealed epithelioid and giant cell granulomas with caseous necrosis (Figure 3), findings strongly suggestive of breast tuberculosis in the appropriate clinical context.

**Figure 3:**
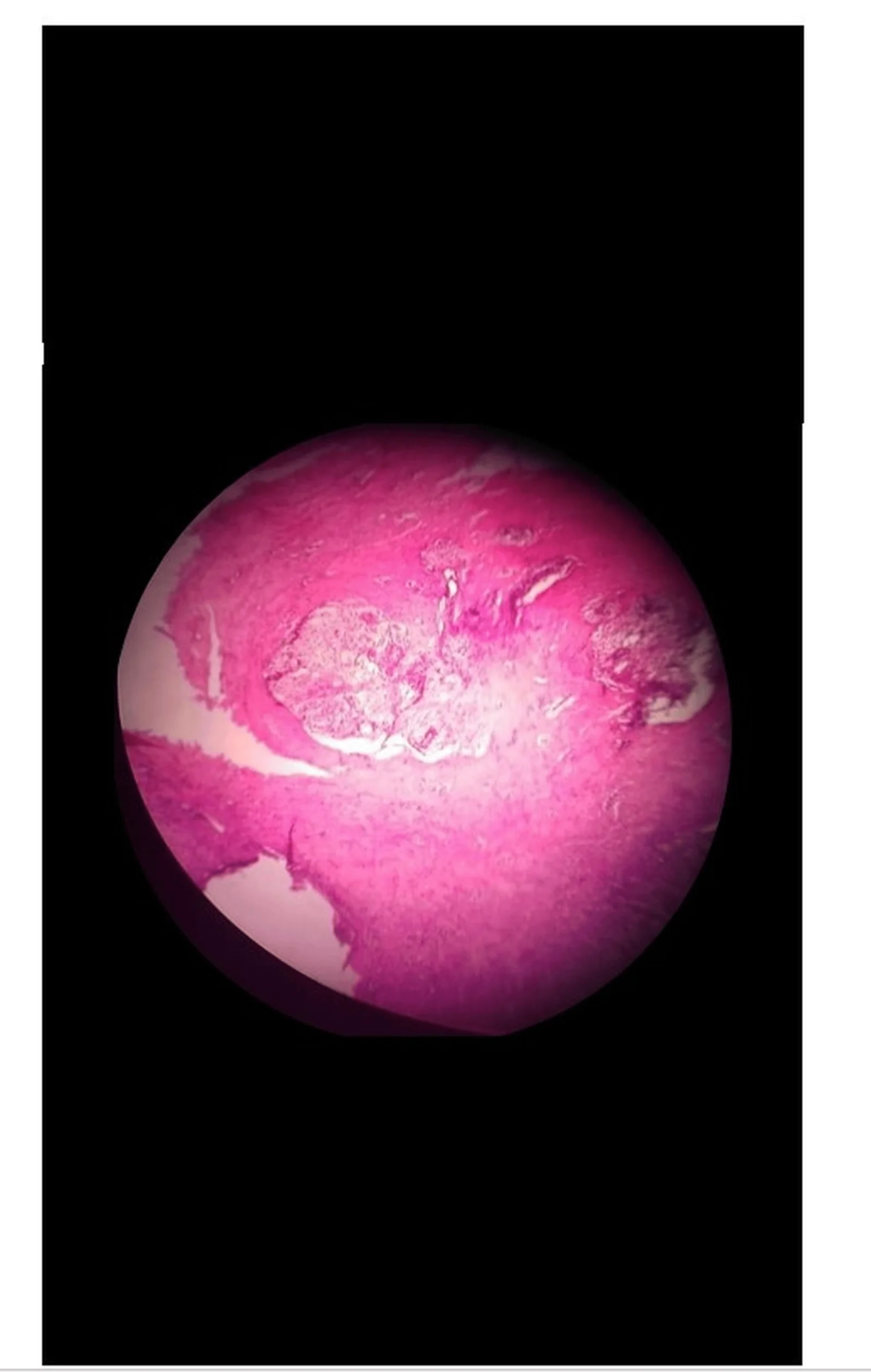
Histopathological findings of breast tuberculosis Histological section showing epithelioid and giant cell granulomas with caseous necrosis, findings strongly suggestive of breast tuberculosis in the appropriate clinical context.

Additional diagnostic tests were performed to evaluate for active pulmonary tuberculosis. Chest radiography was normal. Sputum smear microscopy for acid-fast bacilli (AFB) was negative. The axillary lymph node was not sampled for microbiological or histopathological examination. Microbiological confirmation of *M. tuberculosis* was not obtained from the breast biopsy specimen. However, given the persistent lesion despite antibiotic therapy, associated systemic symptoms and axillary lymphadenopathy, and characteristic histopathological findings, the overall clinical picture was considered strongly suggestive of breast tuberculosis.

The patient received a six-month antituberculous treatment regimen according to the 2RHZE/4RH scheme (isoniazid, rifampicin, pyrazinamide, and ethambutol for two months, followed by isoniazid and rifampicin for four months), with favorable clinical evolution (Figure 1). Percutaneous drainage was performed in conjunction with medical management. The lesion subsequently fistulized and progressively healed under antituberculous treatment.

## Discussion

Breast tuberculosis is a rare form of extrapulmonary tuberculosis, even in endemic countries. Most reported cases have originated from India, although the disease can occur in other high-burden settings [1,2]. The rarity of breast involvement may be explained by the relative resistance of breast tissue to the multiplication of *M. tuberculosis* [3,4]. Young age, female sex, pregnancy, lactation, and multiparity have been reported as potential predisposing factors [1,5]. In the present case, the patient was 31 years old, primiparous, and neither pregnant nor breastfeeding.

Two forms of breast tuberculosis are generally described: primary tuberculosis, in which the breast is the only clinically apparent site of disease, and secondary tuberculosis, in which another tuberculous focus is identified [3,6]. Several routes of infection have been proposed, including hematogenous dissemination, particularly in miliary tuberculosis; lymphatic spread from axillary, mediastinal, or cervical lymph nodes; ductal spread; and, more rarely, direct extension from adjacent structures or inoculation through damaged skin [7,8]. In our patient, the presence of axillary lymphadenopathy associated with extensive breast involvement suggests a possible lymphatic route of dissemination.

Based on its clinical presentation, McKeown and Wilkinson initially classified tuberculous mastitis into five forms: nodular, disseminated or confluent, sclerosing, obliterative, and acute miliary [3,9]. Subsequently, Tewari and Shukla proposed a simplified classification comprising nodular, disseminated, and abscessed forms [1]. This classification reflects the changing clinical spectrum of breast tuberculosis, with sclerosing, obliterative, and acute miliary forms becoming increasingly uncommon, whereas abscess formation appears to be more frequently reported [1,7]. Tuberculous breast abscesses are particularly described in young women and may account for a substantial proportion of reported cases [7,10]. Our patient presented with an abscessed form, initially managed as a pyogenic breast abscess.

The clinical presentation of breast tuberculosis is nonspecific and may closely mimic breast carcinoma or a bacterial breast abscess [1,3]. Patients may present with a breast mass, swelling, pain, skin changes, ulceration, sinus formation, or axillary lymphadenopathy. Certain features should raise suspicion of tuberculosis, particularly recurrent or persistent abscesses that fail to respond to conventional antibiotic therapy, fistulizing axillary lymphadenopathy, and constitutional symptoms such as fever, night sweats, and weight loss [3,10]. In our patient, the persistence of the breast lesion despite conventional antibiotic therapy and the associated axillary lymphadenopathy prompted further investigation and ultimately led to the diagnosis of breast tuberculosis.

A predominance of unilateral disease has been reported, with some studies describing more frequent involvement of the right breast and the upper outer quadrant, possibly because of the proximity of the axillary lymphatic system [7,10]. In our patient, the lesion extensively involved the upper outer quadrant of the right breast and was associated with ipsilateral axillary lymphadenopathy.

Imaging findings are also nonspecific. Ultrasonography is generally the first-line imaging modality and may reveal a hypoechoic, heterogeneous lesion with either well-defined or irregular margins, sometimes associated with posterior acoustic enhancement [3]. Mammography may demonstrate irregular or heterogeneous, poorly defined opacities, sometimes with calcifications, findings that can be mistaken for malignancy [10]. Therefore, imaging alone cannot reliably distinguish breast tuberculosis from breast cancer or pyogenic mastitis.

The definitive diagnosis relies on microbiological and histopathological examination. Demonstration of *M. tuberculosis* by smear microscopy or culture provides direct evidence of infection, while histopathological examination typically demonstrates epithelioid granulomas, Langhans giant cells, and caseous necrosis [1,2]. However, microbiological confirmation may be difficult because the lesions are often paucibacillary. In this context, histopathological examination remains particularly important in establishing the diagnosis and excluding malignancy. In our patient, breast biopsy demonstrated epithelioid and giant cell granulomas with caseous necrosis, confirming the diagnosis of breast tuberculosis.

The treatment of breast tuberculosis is primarily medical and generally follows standard antituberculous therapy, usually administered for six months in uncomplicated disease [1,2]. Surgical intervention is not routinely required and should generally be limited to selected situations, including persistent or large abscesses requiring drainage, diagnostic uncertainty, extensive tissue destruction, or failure of medical therapy [2,7]. In our patient, percutaneous drainage was performed in association with antituberculous treatment, with favorable clinical evolution. Early recognition of breast tuberculosis is therefore important to avoid unnecessary surgical procedures, particularly mastectomy, when the disease can be successfully treated with appropriate medical therapy.

Overall, breast tuberculosis remains a diagnostic challenge because of its ability to mimic both breast cancer and pyogenic mastitis. A high index of suspicion is particularly important in endemic regions and in patients with persistent breast lesions that fail to respond to conventional antibiotic treatment. Histopathological examination, supported when possible by microbiological confirmation, allows an accurate diagnosis and facilitates appropriate medical management.

## Conclusions

Breast tuberculosis, although rare, should be considered in the differential diagnosis of persistent breast swelling, particularly in tuberculosis-endemic countries and in lesions that do not respond to conventional antibiotic therapy. The diagnosis relies mainly on microbiological and histopathological examination. Antituberculous therapy is the cornerstone of treatment, while surgery is reserved for selected cases. Early diagnosis and appropriate treatment generally result in a favorable outcome and may help avoid unnecessary or extensive surgical procedures.
